# People with Intellectual Disabilities Talk About Sexuality: Implications for the Development of Sex Education

**DOI:** 10.1007/s11195-016-9466-4

**Published:** 2016-12-16

**Authors:** D. Schaafsma, G. Kok, J. M. T. Stoffelen, L. M. G. Curfs

**Affiliations:** 1 School of Pedagogical Studies, Fontys University of Applied Sciences, P.O. Box 347, 5600 AH Eindhoven, The Netherlands; 2 Gouverneur Kremers Centrum, Maastricht, The Netherlands; 3 Work and Social Psychology, Maastricht University, Maastricht, The Netherlands; 4 Clinical Genetics, Maastricht University, Maastricht, The Netherlands

**Keywords:** Sexuality, Sex education, Intervention development, Interviews, The Netherlands

## Abstract

Existing sex education programmes have failed in involving people with intellectual disabilities in the development of these programmes. Not involving the target population decreases the likelihood that the sex education programme will be effective. This study was conducted to assess the perspectives of people with intellectual disabilities on several sexuality-related topics. Semi-structured interviews were held with 20 people with intellectual disabilities covering topics such as: sex education, relationships, sex, social media, parenthood and support. The reported frequency of sex education the participants receive is low. Their knowledge regarding sex education is mainly limited to topics such as safe sex, contraception and STI’s and tends to be superficial. Additionally, knowledge on safe sex does not always translate to safe sex behaviour. Finally, relationships are important for most participants; mainly because they don’t want to be alone. Findings from both this study and literature shows that there seems to be a need for high quality sex education. Topics to consider to include are: online relationships, social media and parenthood. It would also be beneficial to focus on sexuality-related skills. Finally, to increase the effectiveness of a sex education programme, it is advisable that a theory-and evidence-based framework, such as Intervention Mapping, is used for its development.

## Introduction

The purpose of sex education programmes is improving sexual health, which is defined as not merely the absence of disease or negative experiences regarding sexuality, but includes positive aspects as well, such as: “the possibility of having pleasurable and safe sexual experiences” [1]. In practice, this means that sexual health is not only about preventing sexually transmitted infections (STI’s), unplanned pregnancies and negative sexual experiences but also about providing people with the means to experience sexuality in a positive way, i.e. through sex education programmes [2]. However, the exact goals of the sex education programmes depend on the needs of the target population and the context in which sex education is provided [3].

Intervention Mapping, a protocol for developing behavior change programmes, states that the development of a behavior change programme, such as a sex education programme, starts with a needs assessment [3]. In this needs assessment problems concerning a certain topic, in this case sexual health, are identified by studying the available literature and, when necessary, by conducting additional research. Developers can search the literature for information on what problems are known in the area of interest and what is effective in improving the situation. Once a problem analysis has been conducted, developers state program goals and create an intervention programme.

A literature review conducted by Schaafsma et al. [4] shows that there is limited information in the scientific literature about what methods are effective for teaching sex education to people with intellectual disabilities. They found that most of the studies in the review did not provide details on what was taught, why it was taught and how it was taught, but mainly focused on whether sex education had an effect on knowledge, attitude or skills. In short, the authors identified a need for more information on what needs to be taught to people with intellectual disabilities with regard to sexuality and how this needs to be taught.

With regard to what needs to be taught, current literature shows that in the area of sexual health, people with intellectual disabilities experience a number of problems. The problems are not necessarily different from people without disabilities, but the percentage of people experiencing these problems is higher. One of the main problems that has been investigated it sexual abuse. People with intellectual disabilities report experiences of sexual abuse [5–8] three times more often than their non-disabled peers [9]. Furthermore, they experience more difficulties in finding, forming and maintaining the relationships they desire so much [6, 10]. These problems may be caused by deficits in sexual knowledge [11–16] and a general lack of social, behavioural and decision skills [17–20].

Environmental factors are also important to consider [3]. Caregivers, parents or paid care staff influence sexual well-being of people with intellectual disabilities: no opportunities are given for sexual experiences or no privacy is provided [11, 21, 22], couples are not allowed to be alone [22] or restrictions are imposed [22, 23]. Paid care staff hardly talk about sexuality with their clients [24, 25] or when they do, it is reactively [10, 24, 26].

Nevertheless, people with intellectual disabilities express a desire for companionship: someone to love, a person that can take care of them or a person to take care of [10–12, 27]. To ascertain that these wishes are met, factors such as knowledge and skills with regard to social behaviour need to be improved with, for example, providing education in the area of sexuality [4, 28].

There are unexplored areas. Social network sites can have a positive impact on the mental well-being of people who are less socially successful in real-life [29]. People with intellectual disabilities are known to have difficulties with social interactions (e.g. interpreting body language) and might benefit from online relationships. Furthermore, the topic of having children is one that is relatively unexplored; most studies on parenthood focus on pregnant women or couples with children [30].

This paper describes an exploratory study to establish the perspectives of people with intellectual disabilities on sexuality-related topics. The goal was to do a needs assessment by identifying problems in the area of sexuality and compare them to the literature. Additionally, relatively unexplored areas, such as social media and parenthood, were investigated. The main topics were sex education, relationships, sex, social media, parenthood, and support. These perspectives were determined by semi-structured interviews with people with intellectual disabilities themselves.

## Methods

### Participants and Recruitment

Twenty participants took part in this study, ten male and ten female. The average age was 28.9 (15–52). Participants were recruited through organizations that provide services to people who are diagnosed with having an intellectual disability. Eight participants lived by themselves and twelve in a group home. Most people living by themselves were over 30; most people living in a group home were under 30 (see Table 1).

**Table 1:** Age of the participants and their living situation

Age	Male	Female	Own^a^	Group^a^
*Number of participants*				
<20	1	4	0	5
20–29	4	2	1	5
30–39	4	1	4	1
40+	1	3	3	1
Mean age	29.2	28.5		

^a^Living environment: own = by themselves, group = in a group home

Participants were recruited through organizations specialized in care for people with intellectual disabilities. We also recruited participants through self-advocacy groups. Most participants receive care from an organization, even when they live outside the organization. Some participants are member of an organization that advocates for rights of people with intellectual disabilities. Potential participants were provided with a leaflet containing information about the interviewer, purpose of the study, confidentiality and data processing. This leaflet was developed with people with intellectual disabilities themselves to increase understandability. People were given time to consider participation. When interested, the researcher would set up an appointment. Interviews took place in an environment where the participant felt most comfortable. Interviews lasted between 30 and 90 minutes.

### Procedure, Ethical Considerations and Interview Topics

**Fig. 1:**
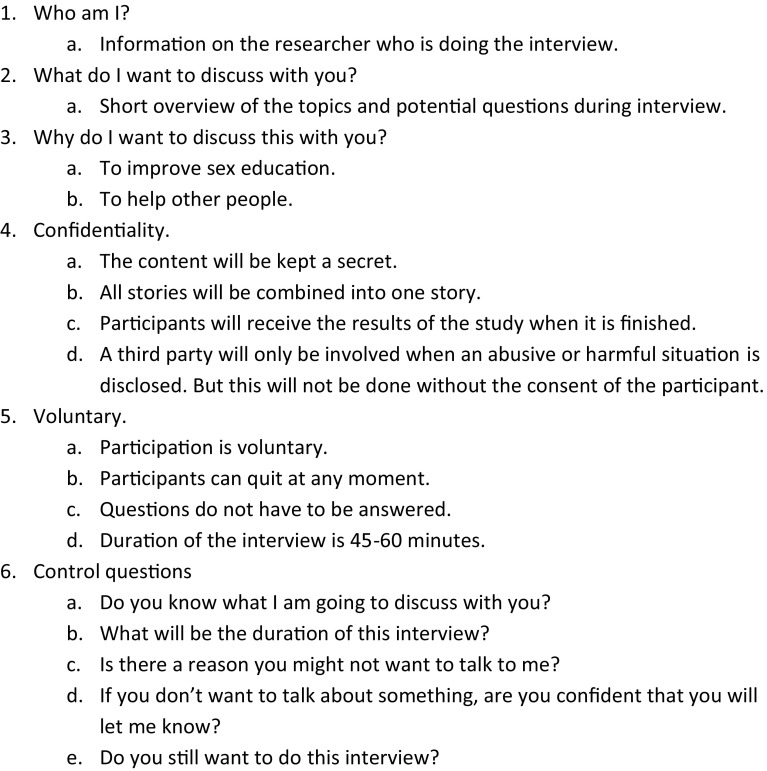
Informed consent procedure

Ethical approval was acquired from the Ethics Committee of the Faculty of Psychology and Neuroscience at Maastricht University. Informed consent was acquired with a procedure developed by Thomas and Kroese [31], including some questions to check the participants’ understanding of the study. For minors (<18 years) participating in this study, consent was provided by their legal guardians. Before the start of the interview, different topics were discussed with the participants, including what would happen in case of disclosure of sexual abuse (see Fig. 1).

This study was aimed at conducting step 1 of the Intervention Mapping protocol [3], a needs assessment. This requires an explorative approach in which different areas concerning sexuality of people with intellectual disabilities are explored to identify potential problems that can be addressed with sex education. Therefore, a semi-structured interview was chosen. Topics were: sex education, relationships, sex, social media, parenthood, and support (see “Appendix”). Social media and parenthood are common topics of discussion, but little is known about what people with intellectual disabilities themselves say about it. Support plays an important role in the lives of people with intellectual disabilities. All interviews were conducted by the first author.

### Data Processing and Analysis

The interviews were recorded, transcribed and then coded using NVivo 9. For analysis we applied a three step approach. In step 1, paragraphs of individual transcriptions were given codes, which were basically short descriptions of the contents of that paragraphs. In step 2, categories were identified from the list of codes. Codes that were similar were merged. The paragraphs related to these codes were merged in NVivo as well. The results was a list of major categories (Table 2) similar to the topics in the topic list that was used during the interview (“Appendix”). Step 3 consisted of identifying subcategories in the major categories (Table 2). The content of the different (sub)categories were further analyzed to identify similarities and differences between participants. Analyses were conducted by the first author.

**Table 2:** A list of major categories and subcategories identified during data analysis

Major categories	Subcategories
Sex education	Sex education yes/no, when and where Type of sex education/material/subjects Thoughts on sex education
Sexual experiences	Experiences Thoughts on sex Knowledge of sex
Relationships	Current relationship Past experiences Needs and wishes Thoughts on relationships
Safe sex	Knowledge and education Own experiences Knowledge on STI’s
Parenthood	Wanting children yes/nog and why? What is needed for raising a child
Social media and internet	None
Negative experiences	None
Homosexuality	None
Influence of the environment	None
Advice for sex education	None

## Results

### Sex Education

Of the 20 participants 19 report to have received sex education: 6 of the young adults at school, others from a staff member, a parent or by reading a book. The older adults from a parent, teacher or a course.

Interviewer (I): Did your parents start talking about it (sex) or did you ask them?

Respondent (R): Uhm, I started and I found it very pleasant. They were really glad that I

started talking about it. (Male, 30–39)

All participants received sex education once or twice during their life. Participants indicated that getting sex education was interesting, fun and nice; however, they also mentioned class members being a bit giggly or ashamed and acted a bit tough.

I: When did you receive sex education?

R: I received sex education last year, but if you would ask me questions about it I wouldn’t know what to say. (Female, <20)

### Topics and Knowledge

Topics mentioned most were safe sex, which included condom-use (20), contraception (10) and STI’s (13). Less mentioned topics were development of the fetus, friendship, pregnancy, female body, loverboys^1^ and boundaries. All participants indicated that safe sex is sex with a condom. They knew that condoms prevent pregnancies and diseases. Six participants also mentioned contraceptives like the pill, and fewer mentioned female condom (2), intra-uterine device (3) or contraceptive injection (2). Asked to name STI’s, they often named HIV and Aids (11), and less frequently Chlamydia (3), Herpes (1), Gonorrhea (1) or genital warts (1).

I: Did they tell you which diseases you can get?

R: Aids, and uhm, HIV. But also other genital diseases that can make your genitals smell bad and stuff. (Female, 20–29)

In general the knowledge on STI’s was poor. When asked: “Do you know when you have an STI?”, most answered that you know when you get tested. Only 3 participants mentioned symptoms like fever, lump on the genitals, itchiness or pain.

Participants also had incorrect or incomplete knowledge, such as not being sure about whether the pill also protects against STI’s or whether the condom protects against STI’s, that when a woman is on birth control a condom is not needed, that you have to use a condom when the woman is on her period even when she is on birth control, and that you can only get an STI from a girl who slept with many boys.

R: But the disease can only occur when the girl has slept with several boys. (Male, 20–29)

Two participants mentioned that teachers or caregivers used materials, such as a broom or a banana for demonstrating condom-use. Other sources of information were books, pictures, videos, dolls of human body and magazines for teenagers.

### Homosexuality

Thirteen participants expressed positive attitudes towards homosexuality: “They are just (normal) people” and “You should let people be (themselves)”. However, two participants indicated it is still a taboo. One man thought homosexuality was strange; saying that his father would not want anything to do with him if he were gay. One woman just didn’t like it; also, because they talk so posh.

R: I have no problems with it (homosexuality) (…) everybody should decide for themselves. If it makes them happy it’s fine by me. (Female, 40+)

One homosexual man explained that it was already a difficult process to accept having an intellectual disability and that being gay made it even more difficult. Eight participants reported to know someone who is gay.

### Relationships

Half of the participants were in a relationship and two were developing a relationship. Two older female participants were married.

One young male participant was looking for long distant relationships on purpose, saying that they are less troublesome. Another young male participant claimed that his girlfriend is giving more attention to other guys, implying she is cheating on him, because she has a large number of male friends on her social network page. One young female participant was in a relationship with a 16-year older male. A young female participant indicated to have a lot of problems in her current relationship, such as disputes, verbal abuse and issues concerning the drug-use of her boyfriend.

I: Does he still do drugs?

R: No, he doesn’t do drugs, although he is still addicted to weed. And I find that really annoying. This is a really, how should I say this, difficult issue in our relationship. (Female, 20–29)

In some previous relationships the environment had a large negative influence, such as parents interfering (3), peers spreading lies about cheating (1), restrictions imposed by staff members (2), or problems in the home environment (1).

I: But the relationship did end?

R: Yes.

I: How did that happen?

R: People were telling stories that were not true.

I: What kind of stories?

R: That I was seeing someone else.

I: And then she broke up with you?

R: Yes.

I: Because she didn’t believe you?

R: No, she didn’t believe me. (Male, 20–29)

Participants met their partner at the organization where they live (3), at work (2), on the internet (1), at a discotheque (1),through a friend (a) or a family member (1). Most of them would like to do fun things with their partner and want their partner to be nice. They usually did not refer to the looks of a partner, but to personal traits: sweet, spontaneous, grateful, respectful, decent, not doing strange things, not doing drugs.

Eight participants find having a relationship important, mainly because they do not want to be alone. Participants want someone to share their life with so that they can be there for one another. Two participants explicitly said being single is no fun; however, one person wanted to be single and another one saw both advantages and disadvantages.

I: Why is a relationship important?

R: You can share something with someone. Now I am just alone. (Male, 20–29)

### Sex

#### Experiences

Fourteen participants reported having experience with sex; seven of them reported using or having used condoms. Four women reported to only use contraception.

I: Did you use condoms?

R: No, I was already on birth control back then. (Female, 40+)

One woman never used condoms, because they should only be used “if you have sex dates”. One man admits that it feels better and more intimate to have sex without. And one gay man always wanted to have safe sex, but it went wrong a few times, resulting in a HIV infection.

#### Beliefs

Positive things that were said about sex were: “sex is pleasure for two”; “sex makes you feel good”. But also: “it’s okay if it doesn’t happen”; “sex belongs within a serious relationship”.

For some participants, sex is not the most important thing, while for others it is. They like it and it feels good, but some point out that sex should be enjoyable for both and that love and respect are also important.

R: It (sex) is some kind of addictive thing, I don’t know.

I: Could you do without?

R: No, I am really honest about that. I could do without, but not for a long period of time. (Male, 30–39)

### Parenthood

Fifteen participants indicated that they wanted or had wanted to have children. Three of them believed they would be good parents; three participants expressed concerns. Younger participants wanted to enjoy life before having any children. Others had concerns regarding being able to raise a child.

R: Taking care of a child 24 h a day. I am not sure whether I want that or whether I am able to do that. Because I also have my own problems (…) I already have enough difficulties with myself, what if the child has the same genes as I have? (Female, 20–29)

When being asked the question “what do you need for raising a child”, most referred to materialistic things: money, a house, food, toys, clothing and diapers (10); most also indicated needing a good job (3) and a good relationship (2).

The interviews gave no indication that the topic of parenthood was seriously discussed with others.

I: Do you ever talk about it (parenthood) with someone?

R: This is actually the first time. (Male, 30–39)

### Internet/Social Media

Social media play a role in the lives of people with intellectual disabilities. Eighteen participants had a profile page on a social network site, such as Facebook or Hyves (Dutch) for keeping in touch with friends. One participant mentioned to have over two hundred friends. Profile sites are at one moment hot and the other moment not. None of the participants used Twitter; three participants mentioned it was too difficult.

R: I have Hyves, but I don’t have Facebook, because it seems that a lot of private data is made public, at least that is what I heard. I don’t know whether it is true or not. And I don’t have Twitter, because I don’t even know how it works. (Male, 30–39)

Other social network sites are used for finding a partner; Badoo was mentioned most (5). Two indicated to have found a girlfriend through such sites; however, one had never met her in real-life and the other relationship ended after 2 days.

Five participants received negative messages or no responses to messages. They all adequately disposed of these unwanted sexually oriented messages by blocking and deleting the sender of the messages.

R: You get messages of people who want to humiliate you. They say racist things, without a reason (…) This happened once (…) Of course I blocked him, so he would never be able to contact me again. (Female, 30–39)

### Negative Experiences and Sexual Abuse

#### Sexual Abuse

Four participants disclosed experiences of sexual abuse. All of them were under 23 and placed out of their home. One male participant reported having been sexually abused up to the age of 18 in boarding school by another boy. The other three cases concerned female participants.

R: I was sexually abused there (boarding school) for 7 years by another boy. He put a knife to my throat and a gun against my head. I went to the staff, but they did nothing. I also went to the police, but they collaborated with the boarding school, and they did nothing as well. I was thinking what now? I was powerless. (Male, 20–29)

#### Inappropriate Touching

One older man had a negative experience on vacation with another man. An older female participant kicked a man in his private parts when he did not listen to her refusal. A younger male participant kicked a girl who tried to force him to have sex, since he saw no other way out.

R: Yes, I mean he wanted all kinds of things from me and I told him not to touch me, but he wouldn’t listen. I then kicked him in his privates and then he had to go to the hospital. (Female, 40+)

#### Other Negative Experiences

One older male participant used a razor for pleasuring himself, causing injuries. He also mentioned women saying that he had a small penis and was not good in bed. Another older male participant had been used by former girlfriends, letting him buy their clothes. He also caught a girlfriend cheating on him in his own apartment. Two younger female participants had experiences with guys harassing them over the telephone.

### Support/Environment

#### Paid Care Staff

All participants received some sort of professional support. Twelve participants lived in institutions receiving support around the clock; eight participants lived by themselves receiving support a few hours a week.

Six participants go to their personal care staff member for support concerning sexuality-related issues. Seven other participants felt less comfortable talking to the staff about sexuality, explaining that “it’s none of their business”; “it would make them (the participant) feel embarrassed”; “they always talk about the same things, safe sex and condom-use”; and “it is a bit scary to ask the staff members”.

R:These staff members are very trustworthy (…) They do everything for me, I only have to ask them. (Male, 20–29)

I: Have you ever talked about sexuality with one of the staff members?

R: Yes I have. But they always talk about the same things, about condom-use and safe sex, they all say the same things. (Female, 20–29)

Four participants experienced staff members’ involvement as interference. One woman was almost forced to divorce her husband, because the support agency felt that it was better. Another reported a female staff member wanting to talk to him about sex, but felt that she had nothing to do with it, since she was “not his mother”. One woman wanted more privacy and the staff not to keep an eye on her and her boyfriend all the time. A girl reported a staff member having an ambivalent attitude towards her relationship with her boyfriend, saying: “It’s OK as long as nothing happens”.

#### Family

Family members are not mentioned very often as a support group. Five participants reported getting support or asking support from family members regarding sexuality-related topics. These can be siblings (4), parents (2) or aunts and uncles (1).

#### Friends

Only one girl talked about sex with her friends. Three participants mentioned to have many friends, while half of the participants indicated to have between 1 and 5 friends. However, they did not mention talking to friends about sexuality-related issues. When asked “who are your friends?” two participants answered with: “What are friends actually?” being unsure whether their friends are real friends. Some never mention friends during the interviews.

Three had negative experiences telling colleagues or friends about their personal lives because personal information was passed on to others.

### Advice for Sex Education by Participants Themselves

We asked participants what would be important to tell someone who has not received sex education. Sixteen participants agreed that safe sex is important; always using a condom, and women using contraception. Fewer of them mention consequences of unsafe sex, STI’s, unplanned pregnancies, and topics on an emotional level: “be sweet to each other”; and “don’t do things that you don’t want to do”.

R: (About sex) They should take it slow and be nice with each other. And if they don’t want to (do it), then don’t (…) It is important that you find it enjoyable. (Male, 30–39)

## Limitations of the Study

The data are somewhat superficial, due to the broad range of topics. Half of the participants are older people who generally live by themselves; while the other half exists of younger people who live in an institution. They also come from very different backgrounds. Young people usually reported problems in the home environment, which is reported less by older participants. It has been found that young adults with intellectual disabilities that have been placed out of their homes run a three times higher risk of being sexually abused than their peers [8]. Finally, we must be careful with generalizing results to other countries, due to differences in support systems for people with intellectual disabilities.

## Conclusions and Recommendations

The goal of this study was to identify problems and needs in the area of sexuality of people with intellectual disabilities, that can be addressed through sex education. This was done by conducting a needs assessment, which is the first step in developing a sex education programme, according to the Intervention Mapping protocol [3].

### Sex Education Topics

The topics of sex education that are mentioned by the participants seem to be limited and focused on safe sex, condom-use, contraception and STI’s; comparable to Löfgren- Mårtenson [32] and Rojas et al. [27]. These topics do not cover the entire area of sexuality. Explanations could be: (1) other topics are not discussed; which is comparable to a non-disabled population [33]; (2) other topics are discussed but that knowledge is not retained; or (3) other topics are not strongly associated with sex education, and therefore not mentioned.

Moreover, participants’ knowledge is superficial. When being asked the question “how do you know you have an STI?”, most answered that you know when you get tested and only few mentioned symptoms. This could be because their education lacks depth or because the information is not retained or too complex.

People with intellectual disabilities express the need to find a partner, mostly because they do not want to be alone; comparable to Lesseliers [22]. The problem is that they report having problems with finding, forming and maintaining relationships [10, 24]; due to a lack of knowledge [11–16], support [25, 34] and skills [17–20]. The relationships reported in this study were not what most people would consider normal healthy relationships. Some have problems assessing their relationship status. It is therefore important in sex education to address finding, forming and maintaining relationships.

Most participants use social media to find contacts, but it is unclear what the quality of those relationships is. Szwedo et al. [29] found that forming online relationships can be beneficial for the mental well-being of people who are less socially accepted or capable in forming meaningful relationships in real-life, because there is more time online to respond to a message and online there is no interference of body language (which is hard to interpret for many people with intellectual disabilities). Online relationships might actually be beneficial for them [10, 24]. However, it is advisable to establish what people with intellectual disabilities expect of such a relationship. Including using social media in sex education, developers would have to be up-to-date on what is “in”. For example, some participants with intellectual disabilities found Twitter too complicated. Additionally, due to the anonymous nature of the Internet, people in general are more prone to negative reactions online [35]; the same goes for people with intellectual disabilities. The participants did express good skills to cope with these issues; however, some might not be so capable. Furthermore, it could be important to have some idea of what they do online, and to clarify the fine line between social network sites and dating sites.

Finally, most participants wanted to have children or had that wish in the past. Parenthood was seldom discussed. One participant talked about it for the first time. People with intellectual disabilities could benefit from learning about the psychological impact of having a baby; since the needs they describe mostly focus on materialistic things, indicating they have no real idea of what it takes raising a child. Tools such as realistic baby dolls might help forming a more accurate image. Furthermore, it would be beneficial to discuss what people with intellectual disabilities mean by having a job, a house and a relationship and whether these requirements are feasible in their situation. According to other studies there is a need for parental skills in four areas: basic bodily needs, skill development, emotional development and social development [30, 36]. Future parents need to learn more about child development; how to stimulate child development through play; to discipline children; recognize when children are at risk; and how to “react to the challenges of adolescence” [37]. Moreover, people working with parents-to-be could ensure they understand the information that is given [30]. Feldman and Case [38] found that some parental skills can be improved with low cost and low tech instructional materials.

### Frequency

The frequency of sex education sessions is low or participants did not remember having received sex education. Continuous sex education is needed to maintain high levels of skills and knowledge [4] meaning that people with intellectual disabilities should receive booster sessions to reactivate the acquired knowledge or skills.

McDermott et al. [39] demonstrated that the amount of sexual knowledge acquired is closely related to the number of instructional sessions. Having an intellectual disability makes it even more difficult to obtain and retain knowledge and skills in just a few sessions. The number of times the participants of this study have received sex education does not seem to be sufficient.

### Determinants and Behaviours

Most participants were positive about homosexuality; some however, were not. Also, less than half of the participants indicated to know someone who is homosexual. Sexual diversity might therefore be an important topic to address, especially since research shows that caretakers are not always aware of the needs of homosexual people with intellectual disabilities [6].

Most of the participants have experiences with sex. When it comes to their attitudes towards sex, some participants say sex is important and some do not. In general, it is more important for most participants to be together with someone than to have sex; in concordance with Lesseliers [22]. Furthermore, some participants shared negative experiences; comparable to other studies [9, 40]. Especially for women, sex is not always pleasurable [41–44]. Women tend to play a rather passive role in sex [42, 43], most sexual acts are geared towards ‘pleasuring the penis’ [43] and some consider it “necessary to do in order to satisfy men and to prevent relationships from deteriorating” [27]. This might explain why many participants find being with someone more important than the act of sex itself.

To improve the sexual health of people with intellectual disabilities, negative experiences could be prevented or at least reduced. Knowledge about people’s own needs, desires and boundaries are important, but skills to protect oneself and perform the correct behaviour are important as well [28]. Many sex education programs focus on knowledge [45], but there are studies showing improved self-protection skills [17, 20, 46–48].

### Environment and Context

Who is responsible and most suitable for teaching sex education? In this study, sex education was taught by parents, staff members and teachers. Some used supportive materials and some did not; however, they did use a mishmash of materials. Not everybody is able to teach sex education, partly due to lack of adequate materials. Research among staff members showed that some have conservative attitudes [21, 24, 49, 50] or, as in this study, have ambivalent attitudes. Rohleder [51] found similar ambivalent attitudes, where staff recognized the need, but also experienced anxiety about the potential harm that sex education might cause. Staff members report a lack of experience in dealing with sexuality [21, 24, 49, 50] and a lack of training [10, 21, 52]. Staff members also usually teach sex education reactively [10, 24, 26] instead of preventively. Parents have even more conservative attitudes than staff members [53].

Educators, whether these are parents, staff members or teachers, should be able to teach sex education properly and cover all important topics. Ideally, educators would use materials proven to be effective; however, in the Netherlands the effectiveness of current materials is unknown [47, 54]. Therefore, educators should use materials they feel comfortable with, while being critical about the content of the materials.

Literature on shared decision making might provide some ideas on the necessary skills, such as developing a partnership, determining the client’s needs and wishes, identifying choices, discussing choices with the client, negotiate a decision and resolve conflicts [55].

Friends are not very important regarding sexuality-related issues [23]. This might be because friends are not always the most trustworthy. But it could also be that friends do not have much knowledge or do not feel comfortable talking about this subject. For many people with intellectual disabilities school seem to be the most important source of information [23].

### Improving Sex Education

Findings from literature, combined with findings from this study give an idea on what could be improved in sex education. Sex education should include a broad range of topics and not exclusively focus on safe sex and prevention of STI’s and unwanted pregnancy. Topics that could be considered are: online relationships, social media and parenthood. It would help when educators receive specific programme goals, specifying what people with intellectual disabilities need to know, do and want [3], something most sex education programmes currently lack [47, 54]. Increasing knowledge is not enough; other determinants, such as skills are important as well [28]. Developers should make sure that programmes have multiple sessions and boosters [39] ensuring maintenance of knowledge and skills.

It would be beneficial if programme developers identified the most ideal context and most suitable target group for sex education. Additionally, they need to identify and reduce potential barriers in order for sex education to be successful [3].

Finally, sex education programmes need to be systematically developed using a theory- and evidence-based protocol, such as Intervention Mapping [3] if developers want the programme to be effective [56–62].

## Appendix: Interview Questions

### Sex Education

- What do you know about sex education?
- What do you think of sex education?
- Have you ever received sex education?
- (If no) Would you like too?
- Where can you find information on sexuality-related matters? (Internet, friends, books?)
- Can you go to someone when you have sexuality-related questions? (Parents, staff members, teachers?)

### Relationships

- Are you in a relationship?
- (If no) Do you want a relationship?
- (If yes) Who is the person you have a relationship with?
- Have you had previous relationships?
- (If yes) How many relationships have you had?
- What do you like to do with your girl/boyfriend? What would you like to do with you girl/boyfriend?
- What is the difference with normal friendship?
- How do you get a girl/boyfriend?
- What do others (parents, staff members) think of you having a relationship?
- Do you find it important to have a relationship?

### Sex

- What is sex? Could you explain it to me?
- What do you think about sex?
- Have you ever had sex?
- Have you ever done something you didn’t like?
- Has you partner ever done something he or she doesn’t like?
- What do you know about homosexuality?
- What is safe sex?
- Do your friends talk about sex? What do they think about sex?
- Is sex important?

### Social Media

- Do you have a profile page on Hyves/Facebook/Twitter/Partyflock etc.?
- Have you ever had an online girl/boyfriend?
- Have you had sex chats (online)?
- Do you own a webcam?
- Has anyone every shown you their private parts or other naked body parts on the Internet?
- Have you ever shown your own private parts or other naked body parts on the Internet?

### Parenthood

- Have you ever thought about having children?
- Would you like to have children?
- What would others think of you having a baby?
- Would you be able to take care of a baby?
- What do you need in order to take care of a baby?

## Footnotes

1 Loverboys are human traffickers who make boys and mostly (underage) girls dependent on them by making these boys and girls think they are in a (loving) relationship. After that, these loverboys force them into prostitution or other legal activities.
